# Association of CRP levels and clinical and radiological outcomes in patients with large-vessel occlusion stroke: A MR CLEAN Registry study

**DOI:** 10.1093/esj/23969873251357134

**Published:** 2026-01-01

**Authors:** Yan Wang, Sven P R Luijten, Daniel Bos, Inge A Mulder, Manon Kappelhof, Willeke F Westendorp, Bart J Emmer, Stefan D Roosendaal, Yvo B W M Roos, Ido R van den Wijngaard, Robert J van Oostenbrugge, Diederik van de Beek, Jonathan M Coutinho

**Affiliations:** Department of Neurology, Amsterdam University Medical Center, Amsterdam, The Netherlands; Department of Radiology and Nuclear Medicine, Erasmus MC University Medical Center, Rotterdam, The Netherlands; Department of Radiology and Nuclear Medicine, Erasmus MC University Medical Center, Rotterdam, The Netherlands; Department of Epidemiology, Erasmus MC University Medical Center, Rotterdam, The Netherlands; Department of Biomedical Engineering and Physics, Amsterdam University Medical Center, Amsterdam, The Netherlands; Department of Neurology, Christchurch Hospital, Christchurch, New Zealand; Department of Neurology, Amsterdam University Medical Center, Amsterdam, The Netherlands; Department of Radiology and Nuclear Medicine, Amsterdam University Medical Center, Amsterdam, The Netherlands; Department of Radiology and Nuclear Medicine, Amsterdam University Medical Center, Amsterdam, The Netherlands; Department of Neurology, Amsterdam University Medical Center, Amsterdam, The Netherlands; Department of Neurology and Radiology, Haaglanden Medical Center, The Hague, The Netherlands; Department of Neurology, Leiden University Medical Center, Leiden, The Netherlands; Department of Neurology, Maastricht University Medical Center, Maastricht, The Netherlands; Department of Neurology, Amsterdam University Medical Center, Amsterdam, The Netherlands; Department of Neurology, Amsterdam University Medical Center, Amsterdam, The Netherlands

**Keywords:** C-reactive protein, large vessel occlusion, endovascular treatment, stroke etiology, acute ischemic stroke

## Abstract

**Introduction:**

Inflammation is important in the pathogenesis of acute ischemic stroke (AIS). The association between CRP and outcomes in patients with large vessel occlusion (LVO) stroke receiving endovascular therapy (EVT) has not been fully elucidated.

**Patients and methods:**

We used data from the MR CLEAN Registry (2014–2017), including LVO-AIS patients with intracranial carotid atherosclerotic disease (ICAD), extracranial carotid atherosclerotic disease (ECAD) or atrial fibrillation (AF). The primary outcome was modified Rankin Scale (mRS) score at 90 days. Secondary outcomes included mRS ⩾3 at 90 days, all-cause mortality, successful recanalization, and symptomatic intracranial hemorrhages. CRP was analyzed both dichotomously (>3.0 vs ⩽3.0 mg/L) and continuously, using multivariable regression adjusted for potential confounders.

**Results:**

Among 865 included patients (ICAD: 286; ECAD: 154; AF: 425), median CRP level was 3.4 mg/L (IQR: 2.0–6.1) and 446 patients had elevated CRP (>3.0 mg/L). AF patients had higher CRP than ICAD and ECAD patients (4.0–3.0–3.2 mg/L, *p* = 0.002). CRP >3.0 mg/L was not associated with mRS in the full cohort (acOR 0.983, 95% CI (0.767, 1.260)) or in any etiological subgroups (ICAD: acOR = 0.968, 95% CI (0.626, 1.496), ECAD: acOR = 1.114, 95% CI (0.617, 2.012), AF: acOR = 0.937, 95% CI (0.653, 1.344)). There was also no association between CRP and any of the other outcomes. When analyzed as a continuous variable, CRP was also not associated with any other outcomes.

**Conclusions:**

We did not observe an association between CRP levels and clinical and radiological outcomes after LVO stroke.

## Graphical Abstract

**Graphical abstract:**
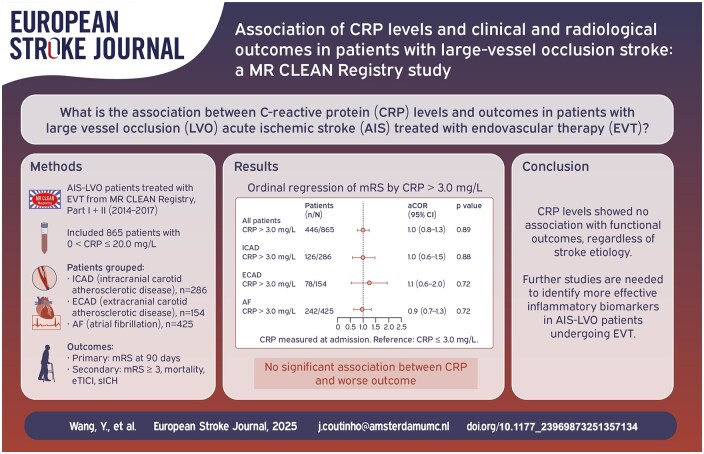

## Introduction

Large vessel occlusion (LVO) is a major cause of acute ischemic stroke (AIS). Endovascular therapy (EVT) has greatly improved the prognosis of patients with LVO stroke, but still around 50% of patients experience disability or die.^1^ Inflammation is believed to play a role both the pathogenesis and recovery after ischemic stroke.^2,3^ In large artery atherosclerosis stroke (LAA), inflammation can lead to atherogenesis, acting as a driver of plaque instability or rupture.^4,5^ In cardioembolic stroke, elevated systemic inflammatory factors (e.g. IL-6) are associated with a poor prognosis.^6–8^

Elevated CRP is associated with stroke risk and poor prognosis.^9–11^ CRP has also been found to be related to recurrence in both patients with intracranial and extracranial atherosclerotic disease.^12,13^ A meta-analysis indicated that elevated CRP is independently associated with a higher risk of stroke and cardiovascular events in patients with atrial fibrillation (AF).^14^

Patients undergoing EVT generally present with larger infarct volumes,^15,16^ which have been associated with a more pronounced inflammatory response.^17^ In addition, many EVT treated patients also receive intravenous thrombolytic therapy. Inflammatory mediators can inhibit endogenous fibrinolysis and enhance procoagulant activity, thereby reducing the efficacy of thrombolysis.^18,19^ The prognostic value of inflammatory factors has not been extensively studied in AIS-LVO patients treated with EVT. Therefore, in the current study, we assessed the association between baseline CRP levels and outcomes in patients with EVT-treated LVO stroke and determined whether this effect differed across subgroups according to stroke etiology.

## Methods

### Study design and population

We used data from the MR CLEAN Registry, a prospective nationwide observational cohort study in the Netherlands. We used data of patients who underwent EVT between March 2014 and November 2017. Comprehensive details regarding the design and procedures of the MR CLEAN Registry have been published.^20^ All imaging was assessed by a core lab whose members were blinded to all clinical information except for occlusion side. CRP measurements are routinely obtained from blood samples collected at admission, before EVT. CRP levels were measured as part of standard-of-care in each participating hospital. While types of assays may differ between hospitals, the results are comparable.

For the current study, we included adult patients (age of 18 years and older) with an LVO of the anterior circulation, confirmed on CTA or MRA (intracranial internal carotid artery (ICA), ICA-terminus (ICA-T), middle cerebral artery (M1/M2), or anterior cerebral artery (A1/A2)). We included patients with an LVO due to intracranial carotid atherosclerotic disease (ICAD), extracranial carotid atherosclerotic disease (ECAD) or atrial fibrillation (AF). Patients with LVO stroke due to other etiologies (e.g. dissection), with multiple causes, or with cryptogenic stroke were excluded. By including only well-defined stroke subcategories, we aimed to reduce heterogeneity and improve our ability to detect differential associations among various subgroups. We further excluded patients with missing data on the 90-day modified Rankin Scale (mRS) score, baseline CRP level, poor Non-Contrast enhanced Computed Tomography quality. We also excluded patients with CRP levels above 20 mg/L, as such a high level is more likely to be related to infection or systemic inflammatory diseases.^21,22^ This exclusion aimed to reduce the possibility of including cases of acute infection or severe systemic inflammatory response, which may affect the association between CRP and stroke outcomes.

ICAD was defined as the presence of intimal calcification in the intracranial ICA on baseline non-contrast computed tomography using a validated scoring method. This method evaluates the circularity, thickness and morphology of intracranial ICA calcification to distinguish intimal from medial arterial layer calcification, with the latter being a phenotype of non-atherosclerotic vascular disease.^23,24^

ECAD was defined as an atherosclerotic stenosis >50% or occlusion of the ICA at the carotid bifurcation (±3 cm) based on baseline CTA.

AF was defined as a history of previous AF or the identification of new-onset AF during hospitalization.

Increased CRP was defined as a CRP level >3.0 mg/L, similar to a previous study.^25^

This retrospective observational study was conducted and reported in accordance with the STROBE (Strengthening the Reporting of Observational Studies in Epidemiology) guidelines. A completed STROBE checklist is provided in the Supplemental Materials.

### Outcome measures

The primary outcome was the 90-day mRS score. Secondary outcomes included dichotomized functional outcome (mRS 0–2 vs 3–6), all-cause mortality, successful recanalization (defined as an extended thrombolysis in cerebral infarction (eTICI) ⩾2B), National Institutes of Health Stroke Scale (NIHSS) score 24–48 h after intervention, duration of procedure, and symptomatic intracranial hemorrhage (sICH, defined according to the Heidelberg Bleeding Classification.^26^)

### Statistical analysis

We examined the association of CRP with outcomes using two approaches. First, we compared patients with an increased CRP (i.e. >3.0 mg/L) to those with a normal CRP (CRP ⩽3 mg/L). Secondly, we evaluated the association between CRP and the predefined clinical and radiological outcomes using CRP as a continuous variable. The association between CRP and the outcome measures was first analyzed in the entire study cohort. Next, we stratified the cohort into three groups according to stroke etiology: ICAD, ECAD, and AF.

For baseline and outcome variables, continuous variables were assessed for normality using the Shapiro–Wilk test and were found to be non-normally distributed. Therefore, they were summarized as medians with interquartile range (IQR) and compared using the Kruskal–Wallis test. Categorical variables were presented as counts and percentages (*n*%), and group comparisons were performed using the Chi-square test.

We used ordinal regression analysis for the mRS on 90 days and the results are presented as odds ratios (OR). Other binary outcomes, including mRS ⩾3 at 90 days, mortality, successful recanalization, and sICH, were analyzed using binary logistic regression, and the results are presented as OR. For all clinical outcome measures, we included age, sex, baseline NIHSS score, pre-stroke mRS, intravenous thrombolysis, systolic blood pressure, occlusion location, and onset-to-groin time as covariates in the model, based on clinical relevance, biological plausibility, and previous studies. Results are presented as adjusted odds ratios (aOR), with adjusted common odds ratios (acOR) specifically used for the mRS ordinal shift analysis. Both unadjusted and adjusted models are provided with 95% confidence intervals (95% CI). Statistical analyses were performed with SPSS software (IBM SPSS Statistics, version 28).

## Results

**Figure 1.**
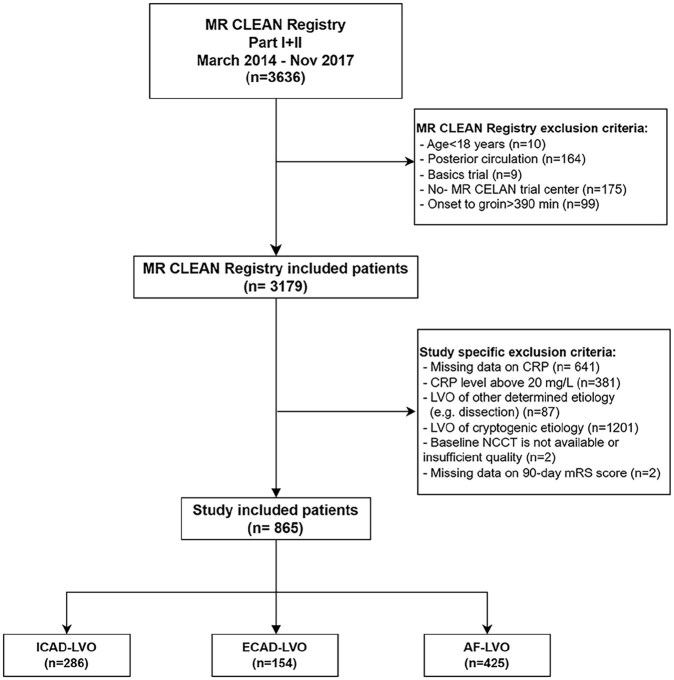
Flowchart for patient selection.

Of 3636 patients in the MR CLEAN Registry, 2771 were excluded (Figure 1). Main reasons for exclusion were missing CRP data, a CRP level above 20 mg/L and stroke of other determined or cryptogenic etiology. Therefore, 865 patients were included in the study: 286 patients with ICAD, 154 patients with ECAD, and 425 patients with AF.

Median age in the study cohort was 74 (66–82) and 51.9% of patients were male (Table 1). Baseline NIHSS score was 15 (11–19). Median CRP level was 3.4 mg/L (2.0–6.1 mg/L) and 446 patients had elevated CRP levels (>3.0 mg/L). Differences between the various subgroups according to cause of the stroke are listed in Table 1. Patients with AF-LVO were older (78 vs 69 vs 73, *p* < 0.001) and less often male (43.8% vs 62.6% vs 54.5%, *p* < 0.001) than patients with ICAD-LVO and ECAD-LVO, respectively. Median CRP levels were slightly higher in the AF-LVO group, compared to the other two groups (4.0 vs 3.0 vs 3.2 mg/L, *p* = 0.002).

**Table 1.** Baseline clinical characteristics.

Characteristic	Full cohort (*n* = 865)	ICAD-LVO (*n* = 286)	ECAD-LVO (*n* = 154)	AF-LVO (*n* = 425)	*p*-value
Age (IQR)	74 (66–82)	69 (61–77)	73 (65–82)	78 (69–85)	<0.001
Male (*n*%)	449/865 (51.9)	179/286 (62.6)	84/154 (54.5)	186/425 (43.8)	<0.001
Baseline NIHSS score (IQR)	15 (11–19)	15 (11–19)	16 (11–19)	16 (11–20)	0.388
Pre-stroke mRS ⩾3 (*n*%)	95/844 (11.3)	24/279 (8.6)	16/147 (10.9)	55/418 (13.2)	0.530
IVT treatment (*n*%)	636/862 (73.8)	246/285 (86.3)	133/153 (86.9)	257/424 (60.6)	<0.001
Systolic blood pressure (IQR)	150 (135–165)	150 (135–165)	155 (143–169)	150 (133–165)	0.026
Diastolic blood pressure (IQR)	82 (72–92)	82 (72–92)	80 (70–90)	84 (73–94)	0.297
Lab results (IQR)					
CRP (mg/L)	3.4 (2.0–6.1)	3.0 (1.7–6.0)	3.2 (1.5–6.1)	4.0 (2.0–7.0)	0.002
Thrombocyte count (10^9/L)	226 (194–277)	236 (201–286)	231 (208–280)	220 (188–270)	0.001
Glucose (mmol/L)	6.9 (5.9–8.2)	7.0 (5.9–8.5)	6.7 (5.8–8.2)	6.8 (6.0–8.1)	0.383
Medical history (*n*%)					
Previous stroke	179/862 (20.8)	51/286 (17.8)	26/153 (17.0)	102/423 (24.1)	0.058
Myocardial infarction	140/851 (16.5)	50/282 (17.7)	27/150 (18.0)	63/419 (15.0)	0.547
Diabetes mellitus	144/858 (16.8)	52/285 (18.2)	25/152 (16.4)	67/421 (15.9)	0.713
Hypertension	493/841 (58.6)	145/279 (52.0)	87/150 (58.0)	261/412 (63.3)	0.012
Hypercholesterolemia	295/835 (35.3)	102/276 (37.0)	52/151 (34.4)	141/408 (34.6)	0.787
Medication use history (*n*%)					
Antiplatelet	291/857 (34.0)	108/284 (38.0)	60/154 (39.0)	123/419 (29.4)	0.021
Oral anticoagulation	40/856 (4.7)	1/285 (0.4)	0/152 (0.0)	39/419 (9.3)	<0.001
Process time, min (IQR)					
Onset-to-groin	199 (150–250)	199 (150–243)	204 (154–260)	197 (150–250)	0.605
Onset-to-reperfusion	254 (201–310)	250 (199–301)	275 (215–330)	250 (198–310)	0.035
Occlusion location (*n*%)					<0.001
Intracranial ICA	53/841 (6.3)	15/278 (5.4)	25/154 (16.2)	13/409 (3.2)	
ICA-T	170/841 (20.2)	61/278 (21.9)	41/154 (26.6)	68/409 (16.6)	
M1	486/841 (57.8)	154/278 (55.4)	72/154 (46.8)	260/409 (63.6)	
M2	123/841 (14.6)	44/278 (15.8)	16/154 (10.4)	63/409 (15.4)	
Other	9/841 (1.1)	4/278 (1.4)	0/154 (0.0)	5/409 (1.2)	
ASPECTS (IQR)	9 (8–10)	9 (8–10)	9 (8–10)	9 (8–10)	0.499
Collateral status (*n*%)					0.151
Absent (grade 0)	47/821 (5.7)	15/271 (5.5)	8/148 (5.4)	24/402 (6.0)	
Poor (grade 1)	298/821 (36.3)	109/271 (40.2)	45/148 (30.4)	144/402 (35.8)	
Moderate (grade 2)	313/821 (38.1)	87/271 (32.1)	61/148 (41.2)	165/402 (41.0)	
Good (grade 3)	163/821 (19.9)	60/271 (22.1)	34/148 (23.0)	69/402 (17.2)	

*p* values are the comparison results among the three subgroups.

**Figure 2.**
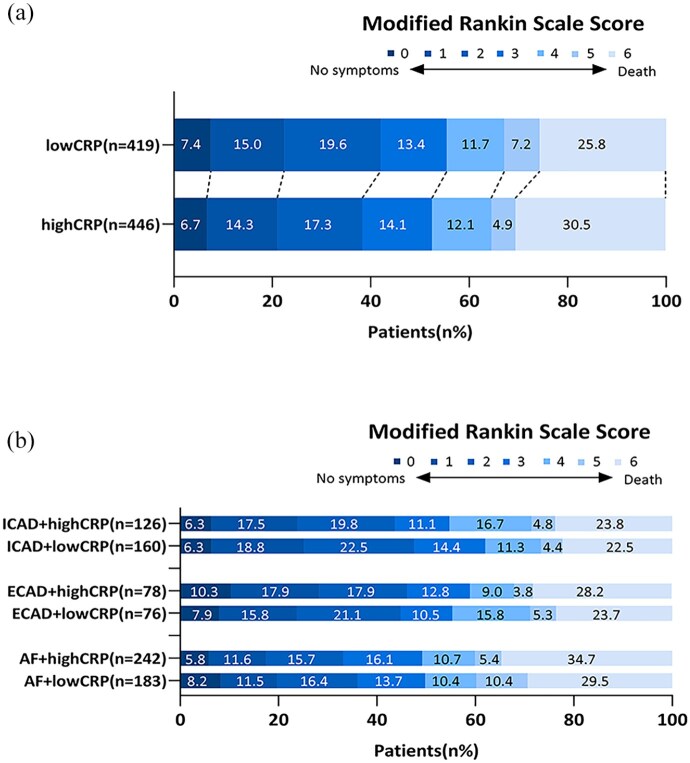
Distribution of patients on the modified Rankin scale at 90 days. (a) The distribution of patients grouped by high and low CRP levels. (b) The distribution of three subgroups based on high and low CRP levels. High CRP: CRP level >3.0 mg/L; low CRP: CRP level ⩽3 mg/L; ICAD: intracranial carotid atherosclerotic disease; ECAD: extracranial carotid atherosclerotic disease; AF: atrial fibrillation.

After adjustment for potential confounders, a CRP level >3.0 mg/L was not associated with a shift on the mRS score (acOR = 0.983, 95% CI (0.767, 1.260)), death or dependency (aOR = 0.988, 95% CI (0.719, 1.358)), all-cause mortality (aOR = 1.016, 95% CI (0.716, 1.443)), successful reperfusion (aOR = 1.057, 95% CI (0.793, 1.410)), or sICH (aOR = 0.801, 95% CI (0.437, 1.467), Table 2 and Figure 2(a)). When performing a subgroup analysis according to stroke etiology, we also did not observe any association between high CRP level and any of the outcomes (Supplemental Tables S1–S3 and Figure 2(b)). Consistently, boxplot analysis showed overlapping CRP distributions between patients with favorable and unfavorable outcomes (*p* = 0.071; Supplemental Figure S1). Furthermore, when analyzed as a continuous variable, higher CRP levels showed a borderline significant association with an increased risk of mortality (aOR = 1.038, 95% CI (1.000, 1.077), *p* = 0.050), but not with any of the other outcomes (Table 3). When stratified according to stroke etiology, we found no association between CRP level as a continuous variable and any of the outcomes (Table 4).

**Table 2.** Outcomes stratified by CRP level.

Outcome	CRP ⩽3.00 mg/L (*n* = 419)	CRP >3.00 mg/L (*n* = 446)	OR (95% CI)	aOR (95% CI)
mRS score at 90 days (IQR)^†^	3 (2–6)	3 (2–6)	0.867 (0.685–1.097)	0.983 (0.767–1.260)
mRS ⩾3 at 90 days (*n*%)^†^	243/419 (58.0)	275/446 (61.7)	1.165 (0.887–1.529)	0.988 (0.719–1.358)
Mortality (*n*%)^†^	108/419 (25.8)	136/446 (30.5)	1.263 (0.938–1.701)	1.016 (0.716–1.443)
Successful recanalization (*n*%)^†^	245/406 (60.3)	260/432 (60.2)	0.978 (0.741–1.290)	1.057 (0.793–1.410)
NIHSS score after 24–48 h (IQR)^‡^	9 (3–15)	11 (4–18)	0.132 (−0.055 to 0.319)	0.073 (−0.107 to 0.252)
Duration of procedure, min (IQR)^‡^	58 (40–83)	60 (35–84)	−0.002 (−0.754 to 0.751)	0.135 (−0.655 to 0.926)
sICH (*n*%)^†^	26/419 (6.2)	22/446 (4.9)	0.784 (0.437–1.407)	0.801 (0.437–1.467)

^†^Binary/ordinal logistic regression. Effect variable is odds ratio. CRP ⩽3 mg/L group was used as the reference group in the analysis.

^‡^Linear regression in CRP >3 mg/L group. Effect variable is Beta.

**Table 3.** Outcomes full cohort with CRP as a continuous variable.

Outcome	Full cohort (*n* = 865)	OR (95% CI)	*p*-value	aOR/acOR (95% CI)	*p*-value
mRS score at 90 days (IQR)	3 (2–6)	0.959 (0.934–0.984)	0.002	0.977 (0.950–1.005)	0.113
mRS ⩾3 at 90 days (*n*%)	518/865 (59.9)	1.037 (1.006–1.070)	0.021	1.014 (0.977–1.053)	0.462
Mortality (*n*%)	244/865 (28.2)	1.059 (1.026–1.092)	<0.001	1.038 (1.000–1.077)	0.050
Successful recanalization (*n*%)	505/838 (60.3)	0.991 (0.962–1.021)	0.556	1.000 (0.969–1.033)	0.979
sICH (*n*%)	48/865 (5.5)	1.007 (0.946–1.073)	0.818	1.013 (0.949–1.082)	0.690

aOR: adjusted odds ratio; acOR: adjusted common odds ratio (used for ordinal logistic regression of mRS shift analysis).

Binary/ordinal logistic regression.

**Table 4.** Outcomes according to stroke etiology..

Outcome	ICAD-LVO (*n* = 286)	ECAD-LVO (*n* = 154)	AF-LVO (*n* = 425)
mRS score at 90 days (IQR)	3 (2–5)	3 (1–6)	4 (2–6)
OR (95% CI)	0.980 (0.934–1.029)	0.966 (0.906–1.029)	0.954 (0.920–0.989)*
acOR (95% CI)	0.978 (0.927–1.033)	1.018 (0.949–1.091)	0.963 (0.926–1.001)
mRS ⩾3 at 90 days (*n*%)	155/286 (54.2)	84/154 (54.5)	279/425 (65.6)
OR (95% CI)	1.003 (0.949–1.060)	1.042 (0.966–1.123)	1.047 (1.001–1.095)^*^
aOR (95% CI)	1.003 (0.933–1.077)	0.978 (0.889–1.076)	1.029 (0.977–1.084)
Mortality (*n*%)	66/286 (23.1)	40/154 (26.0)	138/425 (32.5)
OR (95% CI)	1.040 (0.978–1.105)	1.061 (0.982–1.147)	1.059 (1.016–1.104)**
aOR (95% CI)	1.054 (0.976–1.138)	1.012 (0.924–1.108)	1.052 (1.000–1.107)
Successful recanalization (*n*%)	181/275 (65.8)	83/150 (55.3)	241/413 (58.4)
OR (95% CI)	0.988 (0.933–1.047)	1.006 (0.934–1.082)	0.993 (0.953–1.034)
aOR (95% CI)	0.990 (0.931–1.054)	1.004 (0.926–1.087)	1.005 (0.962–1.050)
sICH (*n*%)	19/286 (6.6)	12/154 (7.8)	17/425 (4.0)
OR (95% CI)	1.001 (0.897–1.118)	1.052 (0.934–1.186)	1.002 (0.906–1.109)
aOR (95% CI)	1.024 (0.909–1.153)	1.050 (0.919–1.199)	1.016 (0.912–1.131)

aOR: adjusted odds ratio; acOR: adjusted common odds ratio (used for ordinal logistic regression of mRS shift analysis).

Binary/ordinal logistic regression.

^*^ *p* < 0.05, **p < 0.01.

## Discussion

In this multicenter observational study, we found no association between CRP levels and clinical or radiological outcomes among patients with a LVO stroke who underwent EVT. This observation was consistent both in the overall cohort and in subgroups according to stroke etiology. These findings suggest that CRP, a widely recognized inflammatory biomarker, may not have prognostic utility in this specific patient population.

Previous studies have linked higher CRP levels at time of admission with poor prognosis in patients with LVO stroke. One single-center study included 676 cases found that patients with CRP levels ⩾5 mg/L at admission had significantly higher adverse functional outcomes (mRS >2) and all-cause mortality within 90 days after EVT compared to patients with lower CRP levels. This effect was particularly evident in patients with concomitant atrial fibrillation.^27^ Another prospective study involving 362 AIS-LVO patients undergoing EVT identified admission high-sensitivity CRP (hs-CRP) levels above 8.255 mg/L were significantly associated with worse 90-day clinical outcomes.^28^ However, in the analysis observing long-term outcomes, this study did not clearly exclude patients with infections or other serious complications, which may affect the accuracy of results. A separate study involving 35 AIS patients showed that CRP levels >0.14 mg/dL were a strong predictor of clinical deterioration at discharge in patients with acute internal carotid artery and middle cerebral artery M1 segment occlusion undergoing recanalization.^29^ However, an important difference between these studies and our study lies in the selection criteria. In the study by Finck et al. patients with very high CRP levels were not excluded, and these patients may be the group with poor prognosis due to concomitant diseases. Instead, we excluded patients with CRP levels greater than 20 mg/L. The study of Uemura et al. had a small sample size, which could increase imprecision of the analysis. In addition, these studies determined cutoff values based on the primary outcome, which increases the risk of a type I error. However, in a single-center cohort of 257 patients with acute ischemic stroke treated with EVT, no significant association was observed between admission hs-CRP levels and clinical outcomes, which supports our findings.^30^

Several studies have shown that elevated CRP levels, especially hs-CRP, are associated with stroke recurrence.^31–34^ Some studies have revealed the prognostic value of hs-CRP in different stroke subtypes. A study showed that hs-CRP levels ⩾3.215 mg/L in LAA stroke patients and hs-CRP levels ⩾1.72 mg/L in small-artery occlusion stroke patients significantly increased the risk of adverse outcomes.^35^ A retrospective study of 4197 patients with minor stroke found patients with both elevated hs-CRP levels (⩾2 mg/L) and intracranial arterial stenosis had a significantly higher risk of recurrent stroke and dependence or death.^36^ Unfortunately, data on long-term risk of stroke recurrence are not available in the MR CLEAN Registry.

Our study has several limitations. First, to ensure homogeneous etiological classification, we excluded patients with cryptogenic stroke or multiple causes. While this approach improved the precision of our subgroup analyses, it also reduced the overall sample size. Second, while the MR CLEAN Registry was a prospective study, the current research question was not planned from inception. As a result, the study should be viewed as retrospective and the results as hypothesis-generating. Finally, the MR CLEAN Registry relies on routine care data, and markers of systemic inflammation beyond CRP, for instance IL-6 and IL-1beta, were not available. Thus, we cannot rule out the possibility that other inflammatory markers are more relevant predictors of post-stroke outcome. Furthermore, we were unable to retrieve data on lipid-related factors that may influence inflammation in stroke, such as low-density lipoprotein and high-density lipoprotein. We could therefore not assess the influence of these factors on the association between CRP and stroke outcomes. It should also be noted that our study focuses on patients with AIS-LVO stroke who underwent EVT. The clinical and radiological outcomes observed in this population, as well as their association with CRP levels, may not be directly applicable to other stroke subtypes, such as small vessel occlusion, or to patients who did not receive EVT.

In conclusion, in this multicenter study, we found no association between CRP levels and clinical or radiological outcomes in LVO stroke patients treated with EVT, neither in the overall cohort, nor in an analysis stratified by stroke etiology. These findings challenge previous reports suggesting that CRP is a strong prognostic marker in ischemic stroke and highlight the need for further research on inflammatory biomarkers that may better predict stroke outcomes in this patient population.

## Supplementary Material

supplementary_files_23969873251357134
